# Cardiovascular Autonomic Neuropathy in Charcot–Marie–Tooth Disease: A Genotype‐Stratified Study of PMP22 Duplication and GJB1 Mutation Carriers

**DOI:** 10.1111/jns.70173

**Published:** 2026-09-27

**Authors:** Kryštof Prášil, Veronika Potočková, Tomáš Nedělka, Alžběta Prášilová, Radim Mazanec

**Affiliations:** ^1^ Department of Neurology, Second Faculty of Medicine Charles University, Motol and Homolka University Hospital Prague Czech Republic; ^2^ Institute of Experimental Medicine Academy of Sciences of the Czech Republic Prague Czech Republic

**Keywords:** cardiovascular autonomic neuropathy, Charcot–Marie–Tooth disease, GJB1, heart rate variability, PMP22

## Abstract

**Background and Aims:**

Data on cardiovascular autonomic neuropathy (CAN) are sparse in Charcot–Marie–Tooth disease. We aimed to determine the prevalence of CAN in CMT1A and CMTX1 and its associations with neuropathy severity and heart rate variability (HRV), compared with age‐ and sex‐matched controls.

**Methods:**

Thirty‐six CMT1A, 24 CMTX1 patients, and 62 controls underwent cardiovascular autonomic testing including the modified Ewing battery (Expiration: Inspiration ratio, 30:15 ratio, Valsalva ratio; complete in 41/62 controls), orthostatic blood pressure measurement, and HRV analysis from 10‐min supine–standing recordings. Abnormality was defined using age‐stratified 2.3rd‐percentile cutoffs. CMT Neuropathy Score version 2 (CMTNSv2) and Survey of Autonomic Symptoms were recorded. Comparisons used Fisher's exact test and the Kruskal–Wallis test with Holm correction; HRV was age‐ and sex‐adjusted.

**Results:**

CAN was present in 30.6% of CMT1A and 33.3% of CMTX1, versus 10.0% of controls (*p* = 0.011). Definite CAN occurred only in CMT1A (11.1%), although the CMT1A–CMTX1 contrast was not significant (*p* = 0.14). The Valsalva ratio was lower in CMT1A than in CMTX1 (median 1.54 vs. 1.89, *p* = 0.001), with abnormal values confined to CMT1A (14.7%). After age and sex adjustment, vagal HRV was reduced in CMT1A but not CMTX1.

**Interpretation:**

CAN affects approximately one‐third of patients with CMT1A and CMTX1, roughly three times the control prevalence. Overall prevalence was similar in the two genotypes, but Valsalva impairment was confined to CMT1A, indicating that similar prevalence does not imply an identical pattern of involvement. Cardiovascular autonomic assessment warrants consideration in these patients, particularly perioperatively.

## 1 Introduction

Charcot–Marie–Tooth (CMT) disease and related inherited neuropathies are the most common hereditary disorders of the peripheral nervous system, with a pooled global prevalence of approximately 18 per 100 000 [1, 2]. They encompass a clinically and genetically heterogeneous group of conditions caused by mutations in over 130 genes, which produce either primary demyelination, primary axonal degeneration, or intermediate forms, all resulting in a length‐dependent, progressive distal sensorimotor phenotype [1]. Among the most prevalent subtypes, CMT1A, which is caused by duplication of the PMP22 gene, is autosomal dominant and demyelinating, accounting for roughly half of genetically confirmed cases [1, 3]. CMTX1, the second most common form, results from mutations in the *GJB1* gene encoding connexin 32, a gap‐junction protein expressed in both Schwann cells and central oligodendrocytes [4].

Cardiovascular autonomic neuropathy (CAN) refers to impairment of autonomic control of the heart and circulation, manifesting clinically as resting tachycardia, exercise intolerance, orthostatic hypotension, and perioperative hemodynamic instability. CAN is best characterized in diabetes mellitus, where it independently predicts cardiovascular morbidity and mortality [5, 6]. The diagnostic standard combines the Ewing battery of cardiovascular autonomic reflex tests [7] with time‐ and frequency‐domain analysis of heart rate variability [8].

Systematic data on cardiovascular autonomic function in CMT1A and CMTX1 are sparse. Early studies in small, mostly genetically unclassified CMT cohorts yielded inconsistent findings: Bird et al. reported cardiac parasympathetic deficit in a subset of patients with type 1 HMSN, whereas Ingall and McLeod found cardiovascular reflexes normal [9, 10]; while Houlden et al., examining pupillary rather than cardiovascular autonomic function, found abnormalities in only 1 of 20 CMT1A and 1 of 18 CMTX1 patients [11]. In the one genetically defined cohort in which cardiovascular reflexes were assessed, Nolano et al. tested 10 patients with CMT1A alongside skin biopsy and interpreted cardiac dysautonomia as infrequent, although no control group underwent the same battery [12]. Lately, a patient‐reported online survey of 669 adults with CMT estimated the prevalence of autonomic symptoms at 65.8% using the Survey of Autonomic Symptoms [13]. A recent controlled comparison across chronic demyelinating neuropathies likewise found a higher overall autonomic symptom burden in 51 patients with CMT1A than in healthy controls, although autonomic function was assessed by questionnaire alone [14]. Perioperative case reports have documented hemodynamic instability attributed to subclinical autonomic dysfunction during general anesthesia, though without genetic characterization that would link such events to specific CMT subtypes [15, 16]. These signals collectively suggest that cardiovascular autonomic involvement in CMT may be both more common and more clinically relevant than is currently appreciated.

To estimate the prevalence of CAN in CMT1A and CMTX1, we performed a cross‐sectional study comparing patients with these genotypes against age‐ and sex‐matched healthy controls, using a standardized protocol of classical cardiovascular autonomic reflex tests, orthostatic blood pressure measurements, and time‐ and frequency‐domain HRV analysis in supine and standing positions.

## 2 Methods and Materials

### 2.1 Study Population

Patients were recruited from the ERN Neuromuscular Center database at the Department of Neurology, Second Faculty of Medicine, Charles University, and at Motol and Homolka University Hospital. The study was approved by the Ethics Committee of the Motol and Homolka University Hospital. All participants entered the study voluntarily and provided written informed consent.

Inclusion criteria were molecularly confirmed hereditary neuropathy (CMT1A and CMTX1) and age 18–67 years. Exclusion criteria were any known alternative cause of CAN, known cardiac arrhythmia, previous myocardial infarction, or implanted pacemaker. No a priori sample size calculation was performed; subgroup sizes reflect the prevalence of each genotype within the registry. Healthy controls were subsequently recruited and frequency‐matched by age and sex.

### 2.2 Phenotyping and Laboratory Assessment

Neuropathy severity was quantified using the CMT Neuropathy Score version 2 (CMTNSv2) [17]; a detailed pharmacological and internal medicine history was supplemented. Autonomic symptom burden was further characterized using the Survey of Autonomic Symptoms (SAS) [18]. Venous blood was drawn to determine renal parameters, hepatic enzymes, glycated hemoglobin, and thyroid hormones, with abnormalities defined against the hospital laboratory's reference ranges.

### 2.3 Standardized Conditions

Functional examinations were performed under standardized conditions: in the morning, after at least 12 h of fasting, with discontinuation of medications affecting heart rate or blood pressure for at least 24 h and abstinence from caffeine and nicotine for at least 12 h prior to testing. Nine participants were taking beta‐blockers (1 CMT1A, 4 CMTX1, and 4 controls); all discontinued ≥ 24 h prior to recording. Nerve conduction studies of the ulnar and radial nerves were performed on the nondominant limb at a minimum dorsal hand temperature of 32°C (Synergy, VIASYS, UK).

### 2.4 Cardiovascular Autonomic Reflex Tests

The modified Ewing battery [19] was performed using the FAN device and software (Schwarzer, Germany), which provides a 4‐lead ECG, respiratory rate monitoring via a thoracic belt, and blood pressure measurement with an upper‐arm cuff [20]. The protocol matched that used by Ziegler et al. [21] to acquire normative reference data, thereby permitting direct comparison with published age‐stratified 2.3rd percentiles.

#### 2.4.1 Lying‐to‐Standing (Orthostatic) Test

After ≥ 5 min of supine rest under standardized conditions (dimmed lighting, quiet room), a 5‐min ECG was recorded in the supine position, followed by a 5‐min recording in the standing position. The 30:15 ratio was calculated as the longest R–R interval among beats 20–40 divided by the shortest R–R interval among beats 5–25 after standing. Blood pressure was measured every minute, supine and after standing, using the same protocol. Orthostatic hypotension (OH) was defined as a mean fall in systolic blood pressure ≥ 20 mmHg or diastolic blood pressure ≥ 10 mmHg, calculated as the difference between the mean of up to four supine readings and the mean of up to four standing readings obtained at 1‐min intervals [22].

#### 2.4.2 Deep Breathing Test

Performed seated, for 3 min at a paced respiratory rate of 6 breaths/min (6 s inspiration/4 s expiration) using a visual breathing guide. From approximately 100 artifact‐free R–R intervals, the cycle with maximal variability was identified, and RR_max_ (expiration) and RR_min_ (inspiration) were used to compute the E–I difference (RR_max_ − RR_min_, ms) and the E:I ratio (RR_max_/RR_min_).

#### 2.4.3 Valsalva Maneuver

Performed seated, with forced expiration against 40 mmHg sustained for 15 s. The Valsalva ratio was calculated as the longest R–R interval after release of the strain divided by the shortest R–R interval during the maneuver.

The cardiovascular protocol was extended mid‐recruitment in controls; all three Ewing tests were included in the protocol for CMT patients throughout.

### 2.5 Heart Rate Variability (HRV)

The 10‐min supine‐and‐standing recording acquired during the orthostatic test (5 min supine + 5 min standing) was additionally used for HRV analysis. Respiration during this acquisition was spontaneous and was not quantitatively analyzed. Participants were instructed to breathe normally and avoid talking. R–R interval series were exported as text files and processed in Kubios HRV Scientific Lite v4.2.0 [23] with beat correction set to “low” threshold; recordings in which > 5% of beats required correction were excluded from HRV analysis. HRV preprocessing was not performed in a blinded manner to genotype; to limit operator‐dependent bias, this beat correction and spectral‐analysis pipeline was prespecified and applied identically to all recordings.

To avoid transition‐related artifacts and the well‐described nonstationary autonomic response in the first 30–60 s after standing, analysis windows were restricted to the first 4 min of the supine recording and the last 4 min of the standing recording. Although this falls marginally below the 5‐min window recommended by the Task Force (1996) [8] for low‐frequency analysis, identical windowing applied across all subjects and conditions ensures that any systematic bias is shared across groups and within‐subject Δ comparisons.

Time‐domain measures (RMSSD, SDNN, pNN50), frequency‐domain measures (LF and HF in absolute units and normalized units, LF/HF ratio), and reactivity measures (Δ = standing − supine) were computed. RMSSD was prespecified as the primary vagal marker, given its lower sensitivity to spontaneous respiratory rate variation than HF spectral power; HF was reported as a confirmatory measure. Spectral measures were log‐transformed before parametric analysis. LF and LF/HF were interpreted as reflecting mixed sympathetic and vagal modulation rather than as direct markers of sympathetic activity. VLF power was not analyzed due to the short recording duration, in line with Task Force recommendations [8].

### 2.6 Classification of CAN

Each cardiovascular Ewing test result (E:I ratio, 30:15 ratio, Valsalva ratio) was classified as normal or abnormal using the age‐stratified 2.3rd percentile cutoffs from Ziegler et al. [21], corresponding to approximately the mean −2 SD of the age‐stratified normative distribution. Patients were staged following Ewing's criteria using all performed tests: *normal* (0 abnormal tests), *early CAN* (1 abnormal test), *definite CAN* (≥ 2 abnormal tests), and *severe CAN* (definite CAN plus orthostatic hypotension) [19].

### 2.7 Statistical Analysis

#### 2.7.1 Primary Analyses

The proportion of patients with abnormal results on each cardiovascular reflex test, and the distribution of Ewing CAN stages, were compared across the three groups (controls, CMT1A, CMTX1) using Fisher's exact test. Orthostatic hypotension prevalence was also compared across groups using Fisher's exact test. Pairwise comparisons with controls (CMT1A vs. controls; CMTX1 vs. controls) were conducted with Holm correction across the two groups. CMTNSv2 was unavailable in 8 of 36 CMT1A (22.2%) and 11 of 24 CMTX1 (45.8%) patients; SAS was unavailable in 7 of 36 CMT1A (19.4%) and 12 of 24 CMTX1 (50.0%) patients; both instruments were complete in all controls. Because of this incomplete ascertainment in CMTX1, within‐genotype correlation analyses involving CMTNSv2 and SAS were restricted to CMT1A. The relationships between CMTNSv2 and CAN stage and between CMTNSv2 and HRV measures were examined using Spearman's rank correlation. Because age may influence both neuropathy severity and HRV, the principal association between CMTNSv2 and supine RMSSD within CMT1A was additionally examined using partial Spearman correlation adjusted for age.

#### 2.7.2 Secondary Analyses

HRV measures were compared between the three groups using Kruskal–Wallis tests, followed by prespecified pairwise Mann–Whitney comparisons (CMT1A vs. controls; CMTX1 vs. controls) with Holm correction across the two contrasts. Supine, standing, and reactivity (Δ = standing − supine) values were analyzed separately. Age‐ and sex‐adjusted comparisons used ordinary least‐squares regression. SAS number of symptoms (NoS) and total impact score (TIS) were compared between CMT patients and controls using Mann–Whitney *U* tests. Within the CMT1A subgroup, SAS scores were correlated with supine HRV measures and with CAN status using Spearman rank correlation. Exploratory direct comparisons between CMT1A and CMTX1 used Fisher's exact test for categorical outcomes and Mann–Whitney *U* tests for continuous measures. Post hoc comparisons of HRV between CAN+ and CAN− participants within each group were performed using Mann–Whitney *U* tests. These exploratory analyses were not adjusted for multiple comparisons.

#### 2.7.3 Excluded Patients

Recordings in which > 5% of beats required correction were excluded from HRV analysis. Patients unable to perform individual tests due to physical limitations were excluded from those specific analyses but retained in all tests they could complete.

#### 2.7.4 Software

Statistical analyses were performed in Python 3.x (scipy, pandas, numpy, statsmodels); figures were generated using Matplotlib. Two‐sided *p* < 0.05 were considered statistically significant.

### 2.8 Artificial Intelligence

The authors declare the use of artificial intelligence tools in the preparation of this manuscript. Claude (Anthropic, claude.ai) was used to assist with literature search and screening, to independently recompute statistical results from the source dataset as a verification step, to check consistency between the manuscript, tables, and figure legends, and to improve the clarity and flow of written text. Grammarly (Grammarly Inc.) was used for grammar checking and language editing. All content generated or modified with the assistance of these tools was reviewed, verified, and approved by the authors, who take full responsibility for the integrity and accuracy of the manuscript. No AI tool was used to generate original scientific content. Only pseudonymized participant data were used; no direct identifiers were provided to any AI tool.

## 3 Results

### 3.1 Study Population

A total of 122 participants were enrolled: 60 patients with genetically confirmed CMT1A and CMTX1 (36 CMT1A, 24 CMTX1) and 62 healthy controls. The sex distribution was 14 male/22 female in CMT1A, 8 male/16 female in CMTX1, and 24 male/38 female in controls (*χ* ^2^ *p* = 0.885). Groups were well matched for age: median 40 years (IQR 30–53) in CMT1A, 45 (34–50) in CMTX1, and 47 (32–56) in controls (Kruskal–Wallis *p* = 0.577). BMI was comparable across groups (medians 23.1–25.6 kg/m^2^; *p* = 0.294). HbA1c differed significantly across groups (CMT1A 35 mmol/mol, CMTX1 34, controls 38; *p* < 0.001), but all individual values fell within the nondiabetic range. Treated hypertension prevalence was similar (controls 19.7%, CMT1A 25.0%, CMTX1 20.8%; *p* = 0.823). Active smoking was less frequent in CMT patients than in controls (controls 22.8%, CMT1A 2.8%, CMTX1 12.5%; *p* = 0.027). Neuropathy severity (CMTNSv2) did not differ between CMT1A and CMTX1 (medians 12 in both; Mann–Whitney *p* = 0.811). Although the history of thyroid disease was more frequent in CMTX1 (4/24), all four affected patients were biochemically euthyroid, and circulating thyroid hormones did not differ across groups (TSH *p* = 0.131, fT4 *p* = 0.281). Demographic and clinical characteristics are summarized in Table 1.

**TABLE 1:** Demographic, clinical, and laboratory characteristics of controls, CMT1A, and CMTX1 participants.

	Control (*n* = 62)	CMT1A (*n* = 36)	CMTX1 (*n* = 24)	*p*
Demographics				
Sex (male/female)	24/38	14/22	8/16	0.885
Age (years)	47 (32–56)	40 (30–53)	45 (34–50)	0.577
Height (m)	1.70 (1.65–1.75) [*n* = 52]	1.70 (1.64–1.74)	1.67 (1.65–1.74)	0.617
Weight (kg)	72 (63–80) [*n* = 51]	68 (59–81)	73 (66–80)	0.433
BMI (kg/m^2^)	23.9 (22.2–26.4) [*n* = 51]	23.1 (21.4–27.4)	25.6 (23.1–27.6)	0.294
Active smoker, *n*/*N* (%)	13/57 (23%)	1/36 (3%)	3/24 (12%)	**0.027**
Treated hypertension, *n*/*N* (%)	12/61 (20%)	9/36 (25%)	5/24 (21%)	0.823
Thyroid disease, *n*/*N* (%)	0/62 (0%)	0/36 (0%)	4/24 (17%)	**< 0.001**
Neuropathy assessment				
CMTNSv2	n/a	12 (10–15) [*n* = 28]	12 (8–16) [*n* = 13]	0.811
SAS number of symptoms	1 (0–3)	3 (2–4) [*n* = 29]	2 (1–5) [*n* = 12]	**< 0.001**
SAS total impact score	1.0 (0.0–6.0)	6.0 (3.0–11.0) [*n* = 29]	6.5 (2.0–14.2) [*n* = 12]	**< 0.001**
Laboratory values				
HbA1c (mmol/mol)	38 (34–40) [*n* = 39]	35 (33–36) [*n* = 28]	34 (33–36) [*n* = 18]	**< 0.001**
Urea (mmol/L)	4.7 (4.2–5.4) [*n* = 42]	4.4 (4.1–5.3) [*n* = 28]	5.1 (4.3–5.9) [*n* = 15]	0.261
Creatinine (μmol/L)	70 (61–80) [*n* = 42]	60 (50–70) [*n* = 28]	72 (66–75) [*n* = 15]	**0.007**
Total cholesterol (mmol/L)	4.8 (4.4–5.5) [*n* = 39]	5.3 (4.5–5.6) [*n* = 28]	5.4 (4.4–6.2) [*n* = 15]	0.684
HDL cholesterol (mmol/L)	1.46 (1.17–1.70) [*n* = 40]	1.60 (1.36–1.83) [*n* = 28]	1.39 (1.17–1.64) [*n* = 15]	0.393
LDL cholesterol (mmol/L)	2.79 (2.32–3.36) [*n* = 40]	2.88 (2.24–3.43) [*n* = 28]	3.31 (2.63–3.82) [*n* = 15]	0.435
Triglycerides (mmol/L)	1.16 (0.79–1.60) [*n* = 40]	0.88 (0.58–1.18) [*n* = 28]	1.07 (0.70–1.32) [*n* = 15]	0.088
TSH (mIU/L)	2.38 (1.25–3.10) [*n* = 42]	1.69 (1.24–2.12) [*n* = 29]	1.59 (1.23–2.25) [*n* = 15]	0.131
fT4 (pmol/L)	14.5 (13.0–15.7) [*n* = 42]	15.1 (13.7–16.5) [*n* = 29]	14.8 (13.5–17.2) [*n* = 15]	0.281

*Note:* Data are median (IQR) for continuous variables and *n*/*N* (%) for categorical variables. *p*: Kruskal–Wallis for continuous, chi‐squared for categorical variables; all across three groups. CMTNSv2 *p*‐value: Mann–Whitney *U* test between CMT1A and CMTX1 only (n/a in controls). [*n* = *x*] or [*x*]: number with available data where ascertainment was incomplete. Bold *p*‐values indicate statistical significance (p < 0.05).

Abbreviations: BMI, body mass index; CMTNSv2, Charcot–Marie–Tooth neuropathy score v2; HbA1c, glycated hemoglobin; SAS, Survey of Autonomic Symptoms.

CMTNSv2 was available in 28 of 36 CMT1A patients (78%) and 13 of 24 CMTX1 patients (54%); SAS data were available in 29 of 36 CMT1A patients (81%) and 12 of 24 CMTX1 patients (50%); both were available in all controls. Within‐genotype correlation analyses involving these instruments were therefore restricted to CMT1A.

CMT patients reported more autonomic symptoms than controls: SAS number of symptoms median 2 (IQR 1–4) in CMT vs. 1 (0–3) in controls (pooled Mann–Whitney *p* < 0.001); total impact score median 6 (3–12) vs. 1 (0–6) (*p* < 0.001).

Two controls lacked sufficient cardiovascular test data for CAN staging and were excluded from staging analyses (CAN‐evaluable *n* = 120). One CMT1A patient was excluded from HRV analyses due to > 5% beat correction in Kubios (HRV‐eligible *n* = 121); this patient was retained in Ewing battery and OH analyses. No patient met the criteria for severe CAN.

### 3.2 Cardiovascular Autonomic Reflex Tests

Per‐test denominators differ across the control group because the cardiovascular protocol was extended mid‐recruitment (see Section 2); CAN staging used all tests performed for each participant.

#### 3.2.1 Per‐Test Abnormality Prevalence

The E:I ratio was abnormal in 22.2% of CMT1A (8/36) and 20.8% of CMTX1 (5/24), compared with 9.5% of controls (4/42); the difference across groups did not reach significance (Fisher exact *p* = 0.267). The 30:15 ratio was abnormal in 11.4% of CMT1A (4/35) and 12.5% of CMTX1 (3/24), compared with 3.3% of controls (2/60; *p* = 0.210). The Valsalva ratio was abnormal in 14.7% of CMT1A patients (5/34) but in none of the CMTX1 patients (0/24) or controls (0/41). The three‐group difference was driven by CMT1A (Fisher exact *p* = 0.007; CMT1A vs. controls Holm‐adjusted *p* = 0.032; Haldane‐corrected OR 15.5, 95% CI 0.82–290.8, wide because no controls had an abnormal Valsalva response). Median test values were comparable between CMT1A and controls (CMT1A 1.54 [1.28–1.71] vs. controls 1.58 [1.42–1.75]), but CMTX1 patients showed a significantly higher median Valsalva ratio than controls (1.89 [1.66–2.10]; KW *p* = 0.002; CMTX1 vs. controls Holm‐adjusted *p* = 0.014). Individual test values, with abnormal results identified against age‐stratified cut‐offs, are shown in Figure 1; pairwise comparisons are presented in Table 2.

**FIGURE 1:**
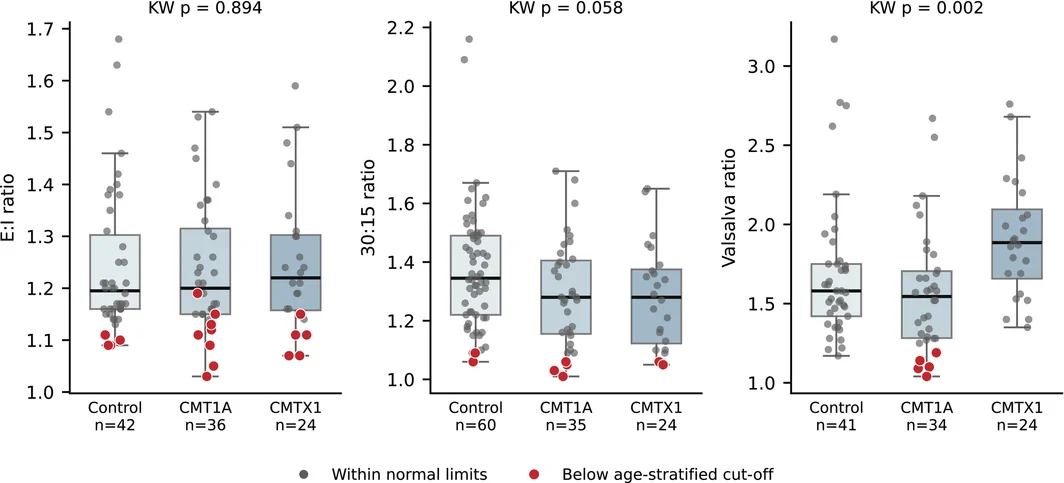
Cardiovascular autonomic reflex test values by group. Boxes show the median and interquartile range, whiskers extend to 1.5 × IQR, and individual participants are plotted as points. Points falling below the applicable age‐stratified 2.3rd‐percentile cut‐off (Ziegler et al. [21]) are shown in red; because the cut‐off is age‐stratified, no single threshold line can be drawn. *p‐*Values are from Kruskal–Wallis tests across the three groups. The number of participants with each available test is given below each box; control denominators differ across panels because the protocol was extended mid‐recruitment (see Section 2). An abnormal Valsalva ratio occurred only in CMT1A (5/34), while CMTX1 patients had a higher median Valsalva ratio than controls (Holm‐adjusted *p* = 0.014).

**TABLE 2:** Prevalence of abnormal cardiovascular autonomic reflex tests, orthostatic hypotension, and cardiovascular autonomic neuropathy (CAN) in each CMT genotype compared with controls.

Test	Genotype	CMT *n*/*N* (%)	Control *n*/*N* (%)	OR (95% CI)	Fisher *p*
E:I ratio abnormal					
	CMT1A	8/36 (22.2%)	4/42 (9.5%)	2.55 (0.74–8.83)	0.207
	CMTX1	5/24 (20.8%)	4/42 (9.5%)	2.41 (0.62–9.40)	0.268
Valsalva ratio abnormal					
	CMT1A	5/34 (14.7%)	0/41 (0.0%)	15.47 (0.82–290.77)	0.016
	CMTX1	0/24 (0.0%)	0/41 (0.0%)	1.69 (0.03–88.12)	1.000
30:15 ratio abnormal					
	CMT1A	4/35 (11.4%)	2/60 (3.3%)	3.34 (0.67–16.65)	0.189
	CMTX1	3/24 (12.5%)	2/60 (3.3%)	3.81 (0.70–20.78)	0.138
Orthostatic hypotension					
	CMT1A	1/36 (2.8%)	7/62 (11.3%)	0.31 (0.05–1.90)	0.251
	CMTX1	0/24 (0.0%)	7/62 (11.3%)	0.15 (0.01–2.75)	0.183
Any CAN (≥ 1 abnormality)					
	CMT1A	11/36 (30.6%)	6/60 (10%)	3.78 (1.30–11.03)	0.014
	CMTX1	8/24 (33.3%)	6/60 (10%)	4.32 (1.35–13.78)	0.020
Early CAN (1 abnormality)					
	CMT1A	7/36 (19.4%)	6/60 (10%)	2.13 (0.68–6.68)	0.226
	CMTX1	8/24 (33.3%)	6/60 (10%)	4.32 (1.35–13.78)	0.020
Definite CAN (≥ 2 abnormalities)					
	CMT1A	4/36 (11.1%)	0/60 (0.0%)	16.75 (0.87–320.97)	0.018
	CMTX1	0/24 (0.0%)	0/60 (0.0%)	2.47 (0.05–127.99)	1.000

*Note:* Each CMT genotype compared with controls using Fisher exact test. Abnormality defined by age‐stratified 2.3rd percentile cutoffs (Ziegler et al. [21]). Fisher *p*‐values are unadjusted; Holm‐adjusted pairwise *p*‐values are reported in the text.

Abbreviations: CAN staging, early (1 abnormal test), definite (≥ 2 abnormal tests); OR, Haldane‐corrected odds ratio with 95% confidence interval.

#### 3.2.2 CAN Staging

Using Ewing criteria, any CAN was present in 30.6% of CMT1A (11/36) and 33.3% of CMTX1 (8/24), compared with 10.0% of controls (6/60; Fisher exact *p* = 0.011). Both CMT genotypes showed significantly elevated CAN prevalence in pairwise comparisons with controls: CMT1A OR 3.78 (95% CI 1.30–11.03), Holm‐adjusted *p* = 0.028; CMTX1 OR 4.32 (95% CI 1.35–13.78), Holm‐adjusted *p* = 0.028. All four cases of definite CAN (≥ 2 abnormal Ewing tests) occurred in CMT1A patients (4/36, 11.1%); none were observed in CMTX1 or controls. No patient met the criteria for severe CAN. The distribution of CAN stage across the three groups is shown in Figure 2; cardiovascular test values, abnormality prevalence, and CAN staging are presented in Table 3. HRV measures in the supine and standing positions are presented in Table 4.

**FIGURE 2:**
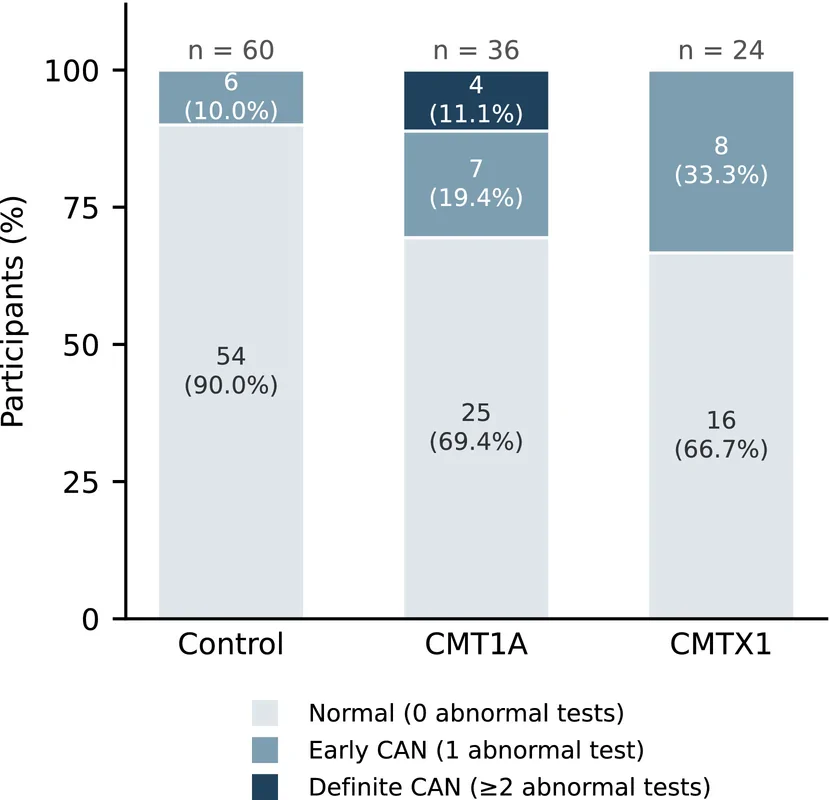
Distribution of cardiovascular autonomic neuropathy (CAN) stage by group. Bars show the proportion of participants at each stage, with counts and percentages shown within each bar. CAN stage was assigned from the three cardiovascular autonomic reflex tests of the modified Ewing battery (E:I ratio, 30:15 ratio, Valsalva ratio), each classified as abnormal when the value fell below the age‐stratified 2.3rd‐percentile cut‐off of Ziegler et al. [21]: Normal = no abnormal test; early CAN = exactly one abnormal test; definite CAN = two or more abnormal tests; severe CAN = definite CAN together with orthostatic hypotension (a fall of ≥ 20 mmHg systolic or ≥ 10 mmHg diastolic on standing, Freeman et al. [22]). Staging used all tests performed for each participant; when fewer than three tests were available, classification was potentially conservative because an abnormality on an unperformed test could not contribute to CAN stage. No participant in any group met criteria for severe CAN, and definite CAN occurred only in CMT1A. Two controls had no evaluable cardiovascular autonomic reflex test and are excluded (control denominator *n* = 60).

**TABLE 3:** Cardiovascular autonomic reflex test values, abnormality prevalence, and CAN staging by group.

	Control (*n* = 60)	CMT1A (*n* = 36)	CMTX1 (*n* = 24)	*p*
E:I ratio				
Median (IQR)	1.19 (1.16–1.30) [*n* = 42]	1.20 (1.15–1.31) [*n* = 36]	1.22 (1.16–1.30) [*n* = 24]	0.894
Abnormal, *n*/*N* (%)	4/42 (9.5%)	8/36 (22.2%)	5/24 (20.8%)	
Valsalva ratio				
Median (IQR)	1.58 (1.42–1.75) [*n* = 41]	1.54 (1.28–1.71) [*n* = 34]	1.89 (1.66–2.10) [*n* = 24]	**0.002**
Abnormal, *n*/*N* (%)	0/41 (0.0%)	5/34 (14.7%)	0/24 (0.0%)	
30:15 ratio				
Median (IQR)	1.35 (1.22–1.49) [*n* = 60]	1.28 (1.15–1.40) [*n* = 35]	1.28 (1.12–1.38) [*n* = 24]	0.058
Abnormal, *n*/*N* (%)	2/60 (3.3%)	4/35 (11.4%)	3/24 (12.5%)	
Orthostatic hypotension				
OH present, *n*/*N* (%)	7/62 (11.3%)	1/36 (2.8%)	0/24 (0.0%)	
CAN staging				
Normal	54 (90.0%)	25 (69.4%)	16 (66.7%)	
Early CAN	6 (10%)	7 (19.4%)	8 (33.3%)	
Definite CAN	0 (0.0%)	4 (11.1%)	0 (0.0%)	
Severe CAN	0 (0.0%)	0 (0.0%)	0 (0.0%)	
Any CAN, *n*/*N* (%)	6/60 (10%)	11/36 (30.6%)	8/24 (33.3%)	**0.011**

*Note:* Continuous test values: median (IQR). Abnormality: *n*/*N* (%) below Ziegler 2.3rd percentile cutoff. [*n* = *x*]: number with available test data. *p*: Kruskal–Wallis test across three groups for continuous values; Fisher's exact test for any CAN prevalence. CAN staging: normal (0 abnormal tests), early (1), definite (≥ 2), severe (definite + OH), any (≥ 1). CAN staging denominators exclude two controls without cardiovascular test data; orthostatic hypotension was assessed in all 62 controls. Bold *p*‐values indicate statistical significance (p < 0.05).

Abbreviation: OH, orthostatic hypotension (ΔSBP ≥ 20 mmHg or ΔDBP ≥ 10 mmHg).

**TABLE 4:** Heart rate variability measures in the supine and standing positions and their reactivity (*Δ* = standing − supine) by group.

Measure	Control (*n* = 62)	CMT1A (*n* = 35)	CMTX1 (*n* = 24)	KW p	CMT1A *p* ^a^	CMTX1 *p* ^a^
*n* (supine/standing)	*62/62*	*35/34*	*24/24*			
Supine						
Mean RR (ms)	903.3 (838.9–1020.2)	898.0 (798.5–989.8)	925.7 (827.9–977.3)	0.485	0.562	0.562
Mean HR (bpm)	66.4 (58.8–71.5)	66.8 (60.6–75.2)	64.8 (61.4–72.5)	0.485	0.562	0.562
SDNN (ms)	36.3 (25.2–51.7)	26.7 (19.9–37.0)	29.4 (24.9–45.5)	0.099	0.074	0.373
RMSSD (ms)	33.2 (20.8–57.3)	24.3 (16.4–34.9)	30.3 (20.9–42.3)	0.189	0.154	0.503
pNN50 (%)	11.0 (1.2–33.7)	3.7 (0.3–15.5)	9.5 (1.8–20.9)	0.199	0.165	0.576
LF power (ms^2^)	592.9 (214.2–1282.6)	302.9 (166.1–542.3)	497.1 (237.4–803.7)	0.128	0.076	0.522
HF power (ms^2^)	412.9 (129.2–1038.8)	155.8 (95.8–578.1)	278.9 (134.2–692.2)	0.158	0.124	0.433
LF (n.u.)	56.8 (44.5–71.3)	51.9 (38.6–78.3)	58.5 (44.4–76.6)	0.975	1.000	1.000
HF (n.u.)	43.2 (28.7–55.5)	48.0 (21.7–61.4)	41.5 (23.4–55.3)	0.973	1.000	1.000
Standing						
Mean RR (ms)	765.3 (690.0–845.4)	748.0 (661.2–835.4)	771.9 (693.5–810.0)	0.605	0.642	0.642
Mean HR (bpm)	78.4 (71.0–87.0)	80.2 (71.8–90.8)	77.7 (74.1–86.6)	0.605	0.642	0.642
SDNN (ms)	28.0 (18.9–41.9)	21.7 (13.2–34.1)	23.3 (19.7–36.6)	0.093	0.065	0.427
RMSSD (ms)	20.8 (13.6–26.7)	15.7 (7.5–23.7)	18.4 (12.2–25.0)	0.081	0.053	0.516
pNN50 (%)	1.5 (0.0–5.3)	0.4 (0.0–3.5)	1.1 (0.0–3.9)	0.269	0.208	0.551
LF power (ms^2^)	494.1 (229.2–1182.9)	315.3 (99.5–658.9)	328.8 (218.9–783.3)	0.085	0.065	0.305
HF power (ms^2^)	119.1 (55.2–257.7)	76.1 (19.4–183.9)	139.9 (41.7–219.7)	0.124	0.084	0.697
LF (n.u.)	79.8 (68.6–87.0)	75.9 (65.5–87.8)	76.2 (65.3–87.7)	0.951	1.000	1.000
HF (n.u.)	20.2 (13.0–31.4)	24.1 (12.1–33.9)	23.8 (12.3 to 34.7)	0.958	1.000	1.000
Reactivity						
Δ SDNN (ms)	−5.7 (−17.8 to 0.2)	−6.2 (−13.7 to 0.7)	−2.2 (−10.8 to 0.9)	0.355	0.751	0.334
Δ RMSSD (ms)	−11.9 (−35.1 to 4.7)	−9.5 (−19.2 to 6.1)	−10.5 (−16.8 to 5.2)	0.774	0.982	0.982
Δ pNN50 (%)	−7.9 (−26.8 to 0.9)	−2.8 (−8.9 to 0.3)	−6.6 (−12.7 to 1.1)	0.362	0.364	0.450

*Note:* Data are median (IQR). All values from 4‐min analysis windows (see Section 2). KW *p*: Kruskal–Wallis test across three groups.

Abbreviations: Δ, standing − supine; HF, high‐frequency power; HR, heart rate; LF, low‐frequency power; n.u., normalized units; pNN50, percentage of successive intervals differing > 50 ms; RMSSD, root mean square of successive differences; RR, R–R interval; SDNN, standard deviation of NN intervals.

^a^ Holm‐corrected pairwise Mann–Whitney *U p*‐value vs. controls (across the two CMT groups).

#### 3.2.3 Sensitivity Analysis Restricted to Complete Cardiovascular Battery

Because the cardiovascular protocol was extended mid‐recruitment, only 41 of 62 controls completed all three Ewing tests, compared with 33 of 36 CMT1A and all 24 CMTX1 patients. The complete‐battery controls were older (median 53 years, IQR 45–58) than the complete‐battery CMT1A (40, IQR 30–54) and CMTX1 (45, IQR 34–51) groups; the sex distributions were 17 male/24 female, 13/20, and 8/16, respectively. Restricting the primary comparison to participants with a complete battery, any‐stage CAN prevalence was 33.3% in both CMT1A (11/33) and CMTX1 (8/24), versus 9.8% in controls (4/41). Both pairwise comparisons remained significant after Holm correction (CMT1A: OR 4.63, 95% CI 1.31–16.31; CMTX1: OR 4.63, 95% CI 1.22–17.59; both Holm‐adjusted *p* = 0.038). Compared with the full available‐case analysis (OR 3.78 for CMT1A and 4.32 for CMTX1), the effect estimates were not attenuated and were slightly larger in the complete‐battery analysis. Definite CAN remained confined to CMT1A (4/33), with no cases among complete‐battery CMTX1 patients or controls.

### 3.3 Heart Rate Variability

#### 3.3.1 Supine and Standing HRV

In unadjusted between‐group comparisons, supine RMSSD did not differ significantly across the three groups (Kruskal–Wallis *H* = 3.33, df = 2, *p* = 0.189): the median value in CMT1A (24.3 ms, IQR 16.4–34.9) was numerically lower than in controls (33.2 ms, IQR 20.8–57.3), while CMTX1 was similar to controls at the group level (30.3 ms, IQR 20.9–42.3). The same direction of difference (CMT1A < controls) was observed for SDNN, pNN50, and absolute HF and LF power, but no Kruskal–Wallis comparison reached significance (all *p* > 0.09), and no pairwise comparison survived Holm correction (CMT1A vs. controls supine RMSSD adjusted *p* = 0.154; SDNN *p* = 0.074; HF power *p* = 0.124). Normalized units (LF n.u.) and the LF/HF ratio did not differ between groups. Standing RMSSD was also lower in CMT1A than controls (15.7 vs. 20.8 ms) but did not reach significance after Holm correction (adjusted *p* = 0.053). Standing HRV measures in CMTX1 did not differ significantly from controls (all Holm‐adjusted *p* > 0.30). Reactivity (Δ) measures of vagal HRV did not differ across groups (all Kruskal–Wallis *p* > 0.35). HRV measures in the supine and standing positions are presented in Table 4.

#### 3.3.2 Vagal HRV by CAN Status

Within each group, participants classified as CAN+ had lower vagal HRV than those classified as CAN−. Supine RMSSD was 20.6 vs. 30.6 ms in CMT1A (*p* = 0.041) and 19.8 vs. 33.0 ms in CMTX1 (*p* = 0.023); the difference was larger in the standing position (CMT1A 10.8 vs. 17.3 ms, *p* = 0.052; CMTX1 12.1 vs. 23.4 ms, *p* < 0.001). The same direction was observed among controls but did not reach statistical significance (supine: 20.1 vs. 33.3 ms, *p* = 0.160). These are post hoc subgroup comparisons (Figure 3).

**FIGURE 3:**
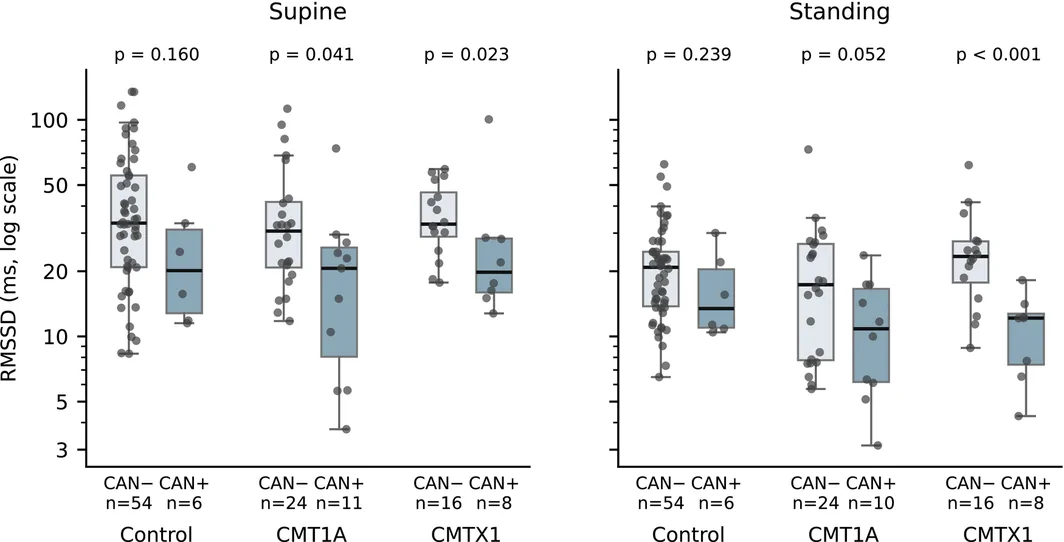
Vagal heart rate variability by genotype and CAN status. RMSSD in the supine (left) and standing (right) 4‐min analysis window, on a logarithmic scale; boxes show median and interquartile range and points are individual participants. CAN status is derived from the staging defined in Figure 2: CAN− = no abnormal cardiovascular autonomic reflex test; CAN+ = one or more abnormal tests, that is, early and definite CAN combined. *p*‐Values are from Mann–Whitney *U* tests comparing CAN+ with CAN− within each group; these are post hoc subgroup comparisons and are hypothesis‐generating. One CMT1A patient was excluded from heart rate variability analyses due to > 5% beat correction, and one further CMT1A standing recording was unavailable; two controls without evaluable testing could not be classified and are not shown. RMSSD, root mean square of successive RR‐interval differences.

#### 3.3.3 Age‐ and Sex‐Adjusted HRV

After adjustment for age and sex using OLS regression, CMT1A showed significant reductions versus controls across multiple HRV measures: supine SDNN (*β* = −11.8 ms, *p* = 0.004), supine RMSSD (*β* = −12.4 ms, *p* = 0.018), supine pNN50 (*β* = −9.4%, *p* = 0.012), log‐transformed supine HF power (*β* = −0.83, *p* = 0.001), and log‐transformed supine LF power (*β* = −0.61, *p* = 0.004). Standing measures showed the same pattern: standing SDNN (*β* = −8.0 ms, *p* = 0.004), standing RMSSD (*β* = −5.5 ms, *p* = 0.038), log‐transformed standing LF power (*β* = −0.77, *p* < 0.001), and log‐transformed standing HF power (*β* = −0.74, *p* = 0.006). CMTX1 showed no significant HRV differences from controls in adjusted analysis (all *p* > 0.08). Adjusted differences from controls across all HRV measures are shown in Figure 4; full unstandardized coefficients are given in Table S1.

**FIGURE 4:**
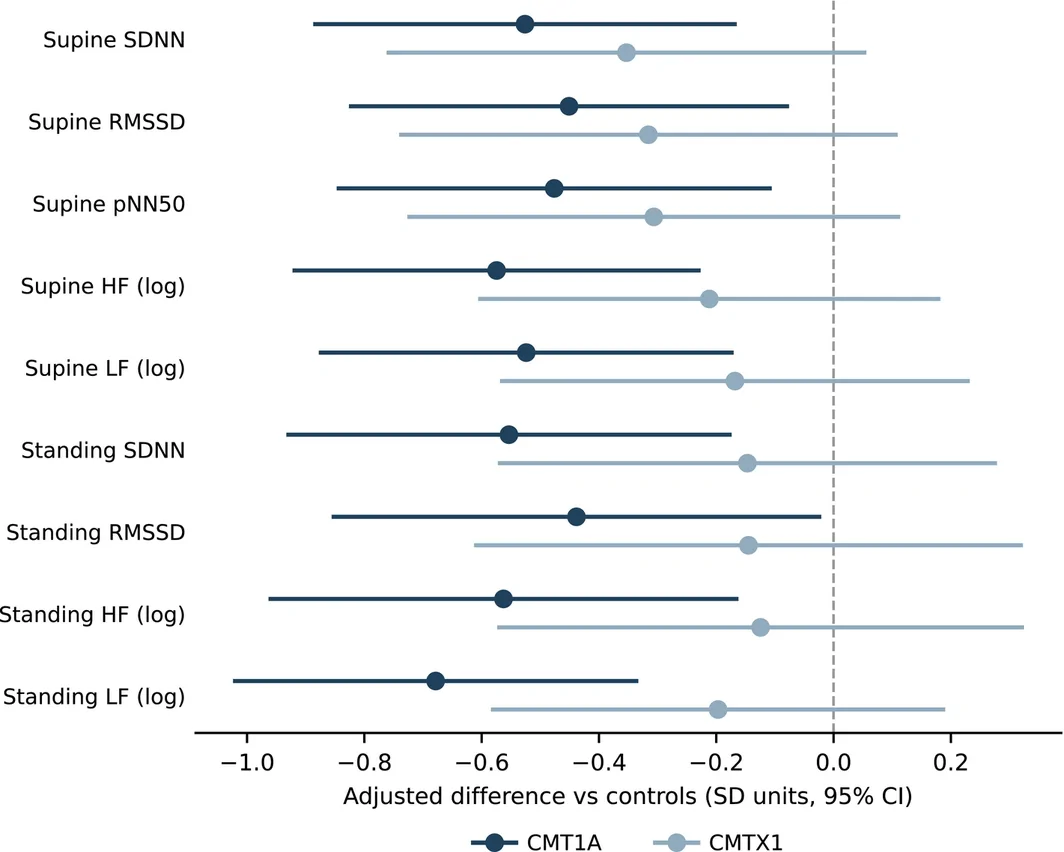
Age‐ and sex‐adjusted differences in heart rate variability relative to controls. Points are regression coefficients for genotype from ordinary least squares models with age and sex as covariates, with 95% confidence intervals; the dashed line indicates no difference from controls. Coefficients are expressed in standard‐deviation units of the respective outcome so that measures recorded in different units can be shown on a common axis; unstandardized coefficients are given in Table S1. Spectral measures were log‐transformed before analysis. Intervals excluding zero correspond to *p* < 0.05: All nine CMT1A coefficients met this criterion, and no CMTX1 coefficient did. Analyses include 121 participants for supine and 120 for standing measures. SDNN = standard deviation of NN intervals; RMSSD = root mean square of successive RR‐interval differences; pNN50 = percentage of successive intervals differing by > 50 ms. HF, high‐frequency power; LF, low‐frequency power.

### 3.4 Direct Comparison Between Genotypes

Any‐stage CAN prevalence was near‐identical in the two genotypes (11/36 vs. 8/24; Fisher exact *p* = 1.00). Definite CAN, observed only in CMT1A, did not differ significantly between genotypes as a dichotomous outcome (4/36 vs. 0/24; *p* = 0.143), nor did the proportion with an abnormal Valsalva ratio (5/34 vs. 0/24; *p* = 0.070); on continuous measures, the Valsalva ratio was significantly lower in CMT1A than in CMTX1 (median 1.54 vs. 1.89; Mann–Whitney *p* = 0.001), whereas the E:I ratio (1.20 vs. 1.22; *p* = 0.700), the 30:15 ratio (1.28 vs. 1.28; *p* = 0.817), and supine RMSSD (24.3 vs. 30.3 ms; *p* = 0.320) did not differ.

### 3.5 Orthostatic Blood Pressure

The change in systolic blood pressure on standing differed significantly across groups (Kruskal–Wallis *p* = 0.014). Controls showed a small median fall in SBP on standing (120.4 → 118.6 mmHg), whereas both CMT groups tended to raise SBP on standing (CMT1A 114.0 → 116.2 mmHg; CMTX1 118.2 → 123.8 mmHg), with both pairwise comparisons reaching significance against controls (Holm‐adjusted *p* = 0.033). Full blood pressure and orthostatic hypotension data are presented in Table 5.

**TABLE 5:** Supine and standing blood pressure and heart rate, orthostatic changes, and prevalence of orthostatic hypotension by group.

	Control (*n* = 62)	CMT1A (*n* = 36)	CMTX1 (*n* = 24)	*p*
Supine (mean of supine readings)				
SBP (mmHg)	120.4 (111.8–131.7)	114.0 (105.9–122.2)	118.2 (109.2–126.4)	**0.045**
DBP (mmHg)	79.2 (74.1–86.5)	74.2 (68.9–81.4)	81.4 (76.9–85.4)	**0.024**
HR (bpm)	64.1 (57.7–71.0)	65.5 (58.7–73.3)	64.2 (59.8–71.6)	0.759
Standing (mean of standing readings)				
SBP (mmHg)	118.6 (109.1–132.0)	116.2 (109.0–130.0)	123.8 (113.1–131.8)	0.490
DBP (mmHg)	86.1 (80.1–92.8)	83.8 (79.9–89.2)	91.2 (87.2–96.6)	**0.005**
HR (bpm)	78.1 (69.9–85.6)	81.8 (70.6–91.2)	76.9 (72.4–88.2)	0.457
Orthostatic change				
ΔSBP (mmHg)	2.2 (−6.1–6.8)	−4.5 (−8.6–2.7)	−4.5 (−12.1–4.3)	**0.014**
ΔDBP (mmHg)	−6.5 (−10.4 to 3.5)	−9.2 (−13.7 to 5.8)	−11.3 (−16.2 to 5.9)	**0.023**
ΔHR (bpm)	12.2 (7.6–19.0)	13.2 (8.3–18.5)	13.4 (9.2–18.8)	0.752
Orthostatic hypotension				
OH present, *n*/*N* (%)	7/62 (11.3%)	1/36 (2.8%)	0/24 (0.0%)	0.169
Minute‐level SBP, supine				
Minute 1	118 (112–135) [*n* = 59]	116 (105–126) [*n* = 32]	120 (111–128)	
Minute 2	121 (112–132) [*n* = 60]	114 (105–119) [*n* = 32]	121 (109–128)	
Minute 3	120 (110–132) [*n* = 61]	112 (104–122) [*n* = 35]	119 (108–128) [*n* = 23]	
Minute 4	118 (112–129)	113 (106–122)	117 (112–122) [*n* = 23]	
Minute‐level SBP, standing				
Minute 6	120 (110–132) [*n* = 59]	118 (110–128) [*n* = 34]	122 (113–135) [*n* = 22]	
Minute 7	120 (108–134) [*n* = 60]	120 (111–131) [*n* = 32]	123 (112–136) [*n* = 23]	
Minute 8	118 (112–132)	120 (109–133) [*n* = 33]	122 (114–134)	
Minute 9	117 (110–132) [*n* = 59]	119 (110–130) [*n* = 33]	126 (111–134)	

*Note:* Data are median (IQR) for continuous variables and *n*/*N* (%) for categorical variables. *p*: Kruskal–Wallis across three groups for continuous variables; Fisher's exact test for OH prevalence. [*n*]: number with available data where incomplete. Bold *p*‐values indicate statistical significance (*p* < 0.05).

Abbreviations: ΔHR, standing HR − supine HR; positive values indicate a rise in heart rate on standing; ΔSBP, supine SBP − standing SBP; positive values indicate a fall in BP on standing. ΔDBP defined analogously; DBP, diastolic blood pressure; HR, heart rate; OH, orthostatic hypotension, defined as ΔSBP ≥ 20 mmHg or ΔDBP ≥ 10 mmHg; SBP, systolic blood pressure.

Under the standard 20/10 mmHg criterion, OH was observed in 7 of 62 controls (11.3%), 1 of 36 CMT1A (2.8%), and 0 of 24 CMTX1 (Fisher exact *p* = 0.169). Per‐subject characteristics of all OH+ subjects, including supine BP, individual standing‐reading BP responses, and heart rate response, are provided in Table S2.

### 3.6 Relationship Between Neuropathy Severity and Cardiovascular Autonomic Function Within CMT1A

#### 3.6.1 CMTNSv2 Correlations

Within CMT1A patients with available CMTNSv2 and HRV data (*n* = 27), neuropathy severity correlated significantly with multiple supine vagal HRV measures: RMSSD (Spearman *ρ* = −0.43, *p* = 0.025), SDNN (*ρ* = −0.43, *p* = 0.027), pNN50 (*ρ* = −0.49, *p* = 0.009), and HF power (*ρ* = −0.45, *p* = 0.018). In an age‐adjusted sensitivity analysis, however, the association between CMTNSv2 and supine RMSSD was markedly attenuated (partial Spearman *ρ* = −0.05, *p* = 0.817). Among Ewing tests, only the 30:15 ratio correlated with CMTNSv2 (*ρ* = −0.40, *p* = 0.040); the E:I ratio (*ρ* = −0.34, *p* = 0.083) and Valsalva ratio (*ρ* = −0.20, *p* = 0.328) did not reach significance. CMTNSv2 did not correlate with ordinal CAN stage (*ρ* = 0.02, *p* = 0.919) or supine heart rate (*ρ* = −0.10, *p* = 0.603). For details, see Table 6.

**TABLE 6:** Spearman correlations of neuropathy severity (CMTNSv2) and autonomic symptom scores (SAS) with cardiovascular autonomic reflex tests and heart rate variability within the CMT1A subgroup.

Measure	CMTNSv2			SAS NoS			SAS TIS		
	*n*	*ρ* (95% CI)	*p*	*n*	*ρ* (95% CI)	*p*	*n*	*ρ* (95% CI)	*p*
Ewing tests									
E:I ratio	27	−0.339 (−0.637 to +0.047)	0.083	28	−0.054 (−0.419 to +0.326)	0.784	28	−0.122 (−0.473 to +0.263)	0.536
Valsalva ratio	26	−0.200 (−0.545 to +0.203)	0.328	27	+0.183 (−0.212 to +0.526)	0.361	27	+0.155 (−0.239 to +0.505)	0.440
30:15 ratio	27	−0.398 (−0.676 to −0.021)	**0.040**	28	−0.074 (−0.435 to +0.308)	0.709	28	−0.206 (−0.538 to +0.181)	0.293
HRV—supine									
RMSSD (ms)	27	−0.432 (−0.697 to −0.062)	**0.025**	28	−0.155 (−0.499 to +0.231)	0.430	28	−0.291 (−0.599 to +0.092)	0.133
SDNN (ms)	27	−0.425 (−0.693 to −0.054)	**0.027**	28	−0.230 (−0.556 to +0.156)	0.238	28	−0.344 (−0.635 to +0.034)	0.073
pNN50 (%)	27	−0.493 (−0.736 to −0.140)	**0.009**	28	−0.130 (−0.480 to +0.255)	0.509	28	−0.287 (−0.596 to +0.096)	0.138
HF power (ms^2^)	27	−0.451 (−0.710 to −0.086)	**0.018**	28	−0.012 (−0.383 to +0.363)	0.953	28	−0.174 (−0.514 to +0.213)	0.375
LF power (ms^2^)	27	−0.181 (−0.525 to +0.214)	0.366	28	−0.457 (−0.709 to −0.101)	**0.014**	28	−0.508 (−0.741 to −0.167)	**0.006**
Mean HR (bpm)	27	−0.105 (−0.466 to +0.287)	0.603	28	+0.210 (−0.177 to +0.541)	0.284	28	+0.176 (−0.211 to +0.516)	0.369
HRV—standing									
RMSSD (ms)	26	−0.309 (−0.622 to +0.089)	0.125	27	+0.019 (−0.364 to +0.396)	0.925	27	−0.077 (−0.444 to +0.312)	0.701
SDNN (ms)	26	−0.235 (−0.571 to +0.167)	0.247	27	−0.141 (−0.494 to +0.253)	0.483	27	−0.231 (−0.562 to +0.163)	0.246
HF power (ms^2^)	26	−0.418 (−0.693 to −0.037)	**0.033**	27	+0.088 (−0.303 to +0.453)	0.664	27	−0.055 (−0.426 to +0.332)	0.784
LF power (ms^2^)	26	−0.241 (−0.574 to +0.162)	0.237	27	−0.085 (−0.451 to +0.305)	0.672	27	−0.226 (−0.558 to +0.169)	0.257

*Note:* Spearman rank correlation within HRV‐eligible CMT1A subgroup (*n* = 35). Per‐row *n* varies with the availability of the predictor (CMTNSv2: 27, SAS: 28) and the measure. 95% CI computed via Fisher *z*‐transformation. Bold *p*‐values indicate statistical significance (*p* < 0.05). Negative *ρ* indicates that worse neuropathy (CMTNSv2) or more autonomic symptoms (SAS) are associated with lower HRV/test values. HRV measures from 4‐min analysis windows; Ewing test values are raw ratios.

Abbreviations: Δ, standing − supine; HF, high‐frequency power; LF, low‐frequency power; n.u., normalized units; pNN50, percentage of successive intervals differing > 50 ms; RMSSD, root mean square of successive differences; SAS NoS, Survey of Autonomic Symptoms, number of symptoms; SAS TIS, total impact score; SDNN, standard deviation of NN intervals.

#### 3.6.2 Graded HRV Reduction Across CAN Stages

Within HRV‐eligible CMT1A patients (*n* = 35), supine vagal HRV declined across Ewing CAN stages: supine RMSSD was 30.6 ms in patients with normal Ewing tests (*n* = 24), 22.9 ms in early CAN (*n* = 7), and 10.3 ms in definite CAN (*n* = 4). Ordered‐rank trend tests were significant for RMSSD (*ρ* = −0.39, *p* = 0.022), SDNN (*ρ* = −0.41, *p* = 0.015), pNN50 (*ρ* = −0.39, *p* = 0.021), HF power (*ρ* = −0.39, *p* = 0.021), and LF power (*ρ* = −0.38, *p* = 0.026) (see Table S3).

#### 3.6.3 SAS–CAN Dissociation

Despite the significantly higher symptom burden reported by CMT1A patients, SAS scores did not correlate with objective measures of cardiovascular function within CMT1A: SAS TIS versus supine RMSSD *ρ* = −0.29 (*p* = 0.133); SAS TIS versus HF power *ρ* = −0.17 (*p* = 0.375); SAS TIS versus CAN stage *ρ* = 0.15 (*p* = 0.450). SAS TIS did not differ between CMT1A patients with and without CAN (median 9 vs. 6; Mann–Whitney *p* = 0.504).

### 3.7 CMTX1 Subgroup Analyses

In an exploratory within‐CMTX1 analysis, the eight patients classified as CAN+ were compared with the 16 CAN− patients. The 16 CMTX1 patients without CAN had HRV comparable to controls (supine RMSSD 33.0 vs. 33.2 ms; HF power 461 vs. 413 ms^2^), whereas the 8 CMTX1–CAN+ patients showed markedly lower values (RMSSD 19.8 ms; HF power 116 ms^2^). The two subgroups differed significantly across multiple measures: supine RMSSD (*p* = 0.023), SDNN (*p* = 0.013), pNN50 (*p* = 0.018), HF power (*p* = 0.013), standing RMSSD (*p* < 0.001), and standing SDNN (*p* < 0.001). Within CMTX1, both the E:I ratio (median 1.1 vs. 1.2; *p* = 0.002) and 30:15 ratio (1.1 vs. 1.4; *p* = 0.004) differed between CAN+ and CAN− subgroups, whereas the Valsalva ratio was identical (1.9 in both; *p* = 0.830). In contrast, among CMT1A–CAN+ patients (*n* = 11), 5 (45.5%) had an abnormal Valsalva ratio versus none of the 8 CMTX1–CAN+ patients (Fisher exact *p* = 0.045) (see Table S4).

## 4 Discussion

In this cross‐sectional, genotype‐stratified study using the modified Ewing battery, orthostatic blood pressure measurement, and time‐ and frequency‐domain HRV analysis, we found that any‐stage CAN was present in approximately one‐third of CMT1A and CMTX1 patients—approximately threefold the prevalence observed in age‐ and sex‐matched healthy controls. Overall CAN prevalence was similar in the two genotypes (*p* = 1.00), but the affected domains differed. The Valsalva ratio was lower in CMT1A than in CMTX1 on direct comparison (*p* = 0.001) and abnormal only in CMT1A. Definite CAN occurred only in CMT1A (4/36), with no cases observed in CMTX1 or controls; however, the direct CMT1A–CMTX1 comparison was not significant (*p* = 0.143). The age‐ and sex‐adjusted vagal HRV reduction reached significance in CMT1A alone, and within CMTX1, a subset showed HRV impairment comparable to CMT1A patients with early CAN. These genotype‐specific observations are exploratory. In CMT1A, supine vagal HRV correlated inversely with CMTNSv2 in unadjusted analyses and declined across Ewing CAN stages; however, the CMTNSv2–RMSSD association was markedly attenuated after adjustment for age, and CMTNSv2 itself was not associated with CAN stage.

### 4.1 Comparison With Previous Studies

Systematic data on cardiovascular autonomic function in CMT remain sparse and inconsistent. Bird et al. found evidence of cardiac parasympathetic deficit in four of seven patients with type 1 HMSN compared with matched controls [9], whereas Ingall and McLeod reported normal cardiovascular reflexes, while documenting impaired extremity sweating consistent with distal sympathetic fiber degeneration [10]. Neither cohort was genetically characterized. Cardiovascular reflexes were assessed in a subgroup of 10 patients with CMT1A by Nolano et al. and interpreted by the authors as showing infrequent cardiac dysautonomia. When we retrospectively classified their published per‐patient results using our staging framework, three of those 10 patients (30%) had at least one abnormal cardiovagal test, close to the 30.6% any‐stage CAN prevalence in our CMT1A group; however, this comparison applies different normative cut‐offs from those used in the original study, and no control group underwent the same battery [12]. The only previous comparison of heart rate variability in CMT against healthy controls studied eight patients of mixed genotype using 24‐h Holter recordings; vagal indices were numerically lower than in controls, but the difference reached significance only for pNN50 [24]. Symptom‐based estimates are considerably higher, with autonomic symptoms reported by 65.8% of 669 patients surveyed using the SAS [13], consistent with the dissociation between symptom burden and objective measures we observed. A recent comparative study of autonomic symptoms across chronic demyelinating neuropathies found a higher total symptom burden in 51 patients with CMT1A than in 125 healthy controls, with significant differences confined to the thermoregulatory and pupillomotor domains; the cardiovascular domain did not separate CMT1A from controls, and no objective cardiovascular testing was performed [14]. In *RFC1*‐related disease, the same Ewing battery demonstrated parasympathetic impairment in 25% and sympathetic impairment in 34% of patients tested despite mild and infrequent autonomic symptoms, the closest methodological parallel to our findings in another genetically defined neuropathy, although the underlying pathology differs [25]. To our knowledge, no previous study has compared cardiovascular autonomic function in genetically defined CMT against matched controls using standardized cardiovascular autonomic reflex testing.

### 4.2 Genotype‐Specific Patterns of Cardiovascular Involvement

Overall CAN prevalence was similar in the two genotypes, but not the pattern: the Valsalva ratio was lower in CMT1A and abnormal only in CMT1A.

In CMT1A, *PMP22* duplication produces uniform demyelination of myelinated peripheral nerve fibers. Both limbs of the cardiovagal reflex arc—the baroreceptor afferents that signal to the brainstem and the preganglionic efferents that mediate the vagal response to the heart—include myelinated fibers, although in neither limb is the population exclusively myelinated. Quantitative electron‐microscopic mapping of the human cervical vagus nerve has shown that approximately 20% of its axons are myelinated; light‐microscopic segmentation of a representative cervical fascicle in the same study showed these myelinated fibers to be bimodally distributed by diameter, with peaks below and above 4 μm [26]. Fiber‐class identity was not determined in that study. A generalized dysmyelinating process could therefore in principle affect either limb of the arc, or both.

Two findings in CMT1A bear on this. Nerve ultrasound has shown the cervical vagus nerve to be enlarged relative to healthy controls (median cross‐sectional area 3 mm [2], range 2–6, vs. 1 mm^2^, range 1–2), enlarged in six of eight patients and varying in proportion to median and ulnar nerve caliber; the authors attributed this to Schwann‐cell proliferation around myelinated vagal axons, the process that produces onion‐bulb pathology in limb nerve [27]. Nolano et al. additionally performed skin biopsy in 20 patients of severity comparable to our cohort (mean CMTNS 10.7), demonstrating loss of sudomotor, vasomotor, and pilomotor innervation, with involvement of small myelinated Aδ fibers attributed to the role of PMP22 in myelination [12]. Structural change in the vagus nerve itself, and involvement of small myelinated cutaneous fibers, have thus both been reported in CMT1A. In a larger skin‐biopsy cohort of 48 patients, intraepidermal nerve fiber density was reduced relative to controls and correlated inversely with CMTNSv2, indicating that cutaneous small‐fiber loss scales with overall neuropathy severity [28].

This framework would be compatible with several of our observations: the inverse correlation between CMTNSv2 and supine vagal HRV is consistent with length‐dependent involvement, although this association did not survive adjustment for age; the graded reduction of HRV across Ewing CAN stages is consistent with, but does not establish, a continuum rather than a binary lesion; and the fact that abnormal Valsalva responses—a maneuver that engages the entire baroreflex arc, from baroreceptor afferent signaling to preganglionic efferent adjustment—occurred only in CMT1A would be consistent with conduction disrupted at multiple points along the reflex loop. Our protocol cannot localize the lesion to the afferent limb, the efferent limb, or both, and the mechanism underlying the Valsalva finding remains unresolved; we therefore offer this framework as a hypothesis consistent with our data rather than as an established mechanism.

Two features of the CMTX1 phenotype in our cohort are noteworthy. First, group‐level HRV did not differ significantly from controls. Exploratory within‐genotype comparisons showed lower HRV measures in the eight patients classified as CAN+ than in the 16 CAN− patients; however, given the small subgroup sizes and exploratory nature of the analysis, these findings should be interpreted cautiously. For detailed variants in the CMTX1 patient cohort (see Table S5).

Second, the Valsalva ratio was completely preserved in CMTX1, including among those patients who met criteria for any‐stage CAN on cardiovascular testing. This dissociation between impaired HRV and an intact Valsalva response argues against a uniform baroreflex denervation in CMTX1. The contrast with CMT1A, where the Valsalva ratio was both lower on direct comparison and abnormal in a subset, is the one genotype difference in our data resting on a significant between‐genotype test. It indicates that similar any‐stage CAN prevalence can conceal differing patterns of involvement; identifying the mechanism will require larger cohorts with adrenergic and afferent‐specific testing.

Taken together, the two genotypes may differ not only in which tests are affected but also in the pattern and degree of cardiovascular autonomic involvement. CMT1A has a relatively uniform molecular basis in recurrent *PMP22* duplications, with substantial inter‐individual variation primarily in disease severity. Accordingly, cardiovascular involvement in CMT1A was graded and, in its most advanced form, multidomain: the E:I ratio was abnormal in all four patients with definite CAN, and all three reflex tests were abnormal in two of them. These four patients were also among the oldest in the cohort (ages 53–66, median 55, vs. a median of 40 years in both the early‐CAN and normal subgroups). Given the cross‐sectional design and the potential influence of age on cardiovascular autonomic measures, we do not interpret this pattern as evidence that definite CAN represents a late, cumulative stage of disease.

In CMTX1, by contrast, 24 patients carried 14 distinct GJB1 variants distributed across multiple Cx32 domains, in both hemizygous males and heterozygous females. The greater genetic and biological heterogeneity of CMTX1 provides a plausible context for the variability observed in cardiovascular autonomic involvement, although our sample is too small to characterize the structure of this heterogeneity.

### 4.3 Orthostatic Blood Pressure Findings

Orthostatic hypotension, defined by the standard 20/10 mmHg criterion, was observed more frequently in controls (7/62, 11.3%) than in CMT patients (1/36 CMT1A; 0/24 CMTX1; three‐group Fisher *p* = 0.169). This unexpected pattern warrants careful interpretation. First, six of the seven OH+ controls were supine‐hypertensive. Applying the higher systolic threshold of 30 mmHg proposed for supine‐hypertensive individuals by Fanciulli and colleagues, while retaining the standard 10 mmHg diastolic criterion, reclassified only one of these six controls as nonhypotensive. Blood‐pressure‐stratified thresholds therefore account for only a small part of the excess OH prevalence in controls, suggesting that other factors discussed below may have contributed [29]. Part of this supine hypertension may itself reflect an anticipatory (“white‐coat”) response in unaccustomed participants, inflating the measured orthostatic change. Second, although participants were encouraged to drink water before testing, fluid intake was not standardized, and variable pretest hydration may have accentuated orthostatic BP variability. Third, measurement variability may also have contributed: examination of individual one‐minute standing readings (Table S2) showed that only three of the seven OH+ controls exhibited a threshold drop sustained across every standing reading, the remainder meeting the mean‐based criterion through single‐reading fluctuation. Fourth, two of the OH+ controls were receiving antihypertensive treatment—one a beta‐blocker—an independent contributor to orthostatic BP dysregulation; although beta‐blockers were discontinued ≥ 24 h before testing, treated hypertension marks underlying blood‐pressure dysregulation independent of any residual drug effect. Finally, three of the seven OH+ controls displayed a paradoxical fall in heart rate on standing, a pattern inconsistent with primary neurogenic OH; under the ΔHR/ΔSBP framework of Norcliffe–Kaufmann and colleagues, several fall into nonneurogenic or technically anomalous categories rather than autonomic‐failure phenotypes [30].

Together, these observations suggest that the apparent excess of OH in controls reflects, at least in part, supine BP dysregulation, antihypertensive‐medication effects, pretest physiological state, and the limitations of mean‐based OH operationalization. More broadly, they reinforce the need for caution in interpreting isolated orthostatic and CAN findings in small cross‐sectional cohorts.

### 4.4 Clinical Implications

The prevalence of objectively measurable CAN—approximately 30% in both CMT1A and CMTX1, though predominantly early‐stage—suggests that cardiovascular autonomic function merits consideration in situations where autonomic compensation is required. Perioperative hemodynamic instability has been documented in case reports of genetically uncharacterized CMT [15, 16]; our findings indicate that a nontrivial proportion of CMT1A and CMTX1 patients carry subclinical cardiovascular autonomic impairment that may remain undetected until challenged by anesthesia, vasoactive medication, or volume shifts. Because clinical cardiovascular events were not ascertained and no longitudinal follow‐up was available, the prognostic significance of these abnormalities remains to be established. CAN was about as common in one genotype as the other, so both warrant the same consideration. What differed was which tests were affected—impaired Valsalva responses occurred only in CMT1A—and whether that reflects a genuinely different kind of autonomic vulnerability will need larger cohorts to establish.

### 4.5 Limitations

Several limitations should be considered. First, the autonomic assessment in this study was predominantly cardiovagal. Adrenergic function was evaluated only through intermittent cuff‐based orthostatic blood pressure measurement, which is sufficient to identify orthostatic hypotension but does not characterize broader sympathetic function; beat‐to‐beat pressure monitoring and tilt‐table testing were unavailable. No sudomotor, gastrointestinal, genitourinary, or pupillary testing was performed. The prevalence figures therefore quantify CAN specifically and cannot be extrapolated to generalized autonomic neuropathy.

Second, the cardiovascular protocol was incomplete in a subset of controls owing to a mid‐study protocol extension; 41 of 62 controls completed all three Ewing tests. CAN stage may have been underestimated because abnormalities on unperformed tests could not contribute to classification. This limitation is particularly relevant to comparisons with controls. In the sensitivity analysis restricted to participants who completed all three tests, the prevalence contrast was not attenuated; incomplete testing may nonetheless have led to underestimation of CAN stage in individual controls.

Third, respiration was neither paced nor recorded during the 10‐min supine–standing acquisition. HF power varies with both breathing frequency and tidal volume, and at respiratory rates below approximately 9 breaths/min, respiratory‐driven variability shifts out of the HF band into the LF band; unmeasured differences in spontaneous breathing pattern could therefore contribute to the HF and LF differences we observed. RMSSD, which is less sensitive than HF power to the frequency at which respiratory variability occurs, was prespecified as the primary vagal measure and showed the same group differences as HF power; the two ranked participants nearly identically (supine Spearman *ρ* = 0.955), arguing against band displacement as the source of our findings. A between‐group difference in respiratory amplitude would affect RMSSD and HF alike and cannot be excluded. This is a substantive limitation, and future studies should record respiration throughout HRV acquisition or use paced breathing.

Fourth, HRV analysis windows were 4 min rather than the 5‐min window recommended by Task Force [8], which specifies at least 10 cycles of the lowest frequency analyzed. Our window provides approximately 36 cycles of the slowest HF component (0.15 Hz) but only 9.6 of the slowest LF component (0.04 Hz), so time‐domain measures and HF are adequately sampled, whereas individual LF estimates are less stable. Because identical windowing was applied across all subjects and conditions, the loss of LF precision is shared across groups and within‐subject comparisons; such error adds scatter and works against detecting a difference rather than creating one. LF was a secondary measure, and no finding here rests on LF alone. The shorter window was accepted deliberately: a full 5‐min standing segment would have included the first minute after standing, when the heart rate response is still unsettled. Finally, standing blood pressure was measured with the arm dependent and unsupported, which may increase measured standing pressure; the orthostatic hypotension prevalences reported here may therefore be underestimated, in all three groups alike.

Fifth, thyroid disease was more prevalent in CMTX1 than in the other groups. Because all affected patients were biochemically euthyroid and thyroid hormone levels did not differ between groups, clinically significant confounding of the HRV comparisons is unlikely.

Sixth, no a priori sample size calculation was performed; subgroup sizes reflect the prevalence of each genotype within the source registry. The consequences are greatest in CMTX1, where 24 patients yielded no abnormal Valsalva responses and no cases of definite CAN; since 0 of 24 is compatible with a true rate as high as 14%, the absence of these findings cannot be taken as evidence that the phenotype does not occur. The CMTNSv2–HRV correlation in CMT1A was assessed in 27 patients with complete data; it was significant in unadjusted analysis but was markedly attenuated after adjustment for age, indicating that the unadjusted association may at least partly reflect age. The same limitation applies to the unadjusted trend in HRV across CAN stages, in which the four patients with definite CAN were also the oldest. SAS data were available in 81% of CMT1A but only 50% of CMTX1 patients, limiting within‐CMTX1 symptom analyses. The CMTX1 subgroup comparisons are based on 8 CAN+ versus 16 CAN− patients and require confirmation in larger genotyped cohorts.

Finally, the cross‐sectional design precludes inference about temporal progression of CAN within individual patients. Longitudinal follow‐up of this cohort, with particular attention to the CMTX1 patients with CAN and to those CMT1A patients with early‐stage CAN, will be required to determine whether cardiovascular involvement progresses in parallel with somatic neuropathy or follows an independent trajectory.

## Conflicts of Interest

The authors declare no conflicts of interest.

## Supporting information


**Table S1:** Age‐ and sex‐adjusted differences in heart rate variability relative to controls: unstandardized regression coefficients for each CMT genotype.
**Table S2:** Individual characteristics of participants meeting the 20/10 mmHg criterion for orthostatic hypotension, including supine blood pressure, standing blood pressure responses, and heart rate response.
**Table S3:** Supine heart rate variability across Ewing CAN stages within the heart‐rate‐variability‐eligible CMT1A subgroup, with tests for monotonic trend.
**Table S4:** Cardiovascular autonomic reflex test values and heart rate variability in CMTX1 patients with and without CAN.
**Table S5:** *GJB1* variants, CAN staging, and cardiovascular autonomic findings in individual CMTX1 patients.

## Data Availability

The data that support the findings of this study are available from the corresponding author upon reasonable request.
