# Supplementary material for: Cardiovascular Autonomic Neuropathy in Charcot–Marie–Tooth Disease: A Genotype‐Stratified Study of PMP22 Duplication and GJB1 Mutation Carriers
Source: J Peripher Nerv Syst. 2026 Sep 27;31(4):e70173. doi: 10.1111/jns.70173 (PMC13616590; doi:10.1111/jns.70173)
Supplement: Supplementary file 1 [file JNS-31-0-s001.docx]

# Supplementary Table S1

| **Measure** | **CMT1A vs Control** | | | **CMTX1 vs Control** | | | **n** |
| --- | --- | --- | --- | --- | --- | --- | --- |
|  | **β** | **SE** | **p** | **β** | **SE** | **p** |  |
| **Supine** | | | | | | | |
| SDNN, ms | -11.80 | 4.05 | **0.004** | -7.92 | 4.59 | 0.087 | 121 |
| RMSSD, ms | -12.38 | 5.15 | **0.018** | -8.66 | 5.84 | 0.141 | 121 |
| pNN50, % | -9.42 | 3.67 | **0.012** | -6.07 | 4.16 | 0.148 | 121 |
| LF power, ms² (log) | -0.61 | 0.21 | **0.004** | -0.20 | 0.23 | 0.403 | 121 |
| HF power, ms² (log) | -0.83 | 0.25 | **0.001** | -0.31 | 0.29 | 0.285 | 121 |
| Mean HR, bpm | 2.38 | 2.18 | 0.278 | 0.79 | 2.48 | 0.751 | 121 |
| **Standing** | | | | | | | |
| SDNN, ms | -8.01 | 2.75 | **0.004** | -2.13 | 3.08 | 0.492 | 120 |
| RMSSD, ms | -5.53 | 2.64 | **0.038** | -1.83 | 2.96 | 0.537 | 120 |
| LF power, ms² (log) | -0.77 | 0.20 | **<0.001** | -0.22 | 0.22 | 0.312 | 120 |
| HF power, ms² (log) | -0.74 | 0.26 | **0.006** | -0.16 | 0.29 | 0.581 | 120 |
| Mean HR, bpm | 3.62 | 2.39 | 0.133 | 2.68 | 2.71 | 0.324 | 120 |

HRV measures were regressed on age, sex, and group membership (CMT1A or CMTX1); controls served as the reference category.

β = regression coefficient (adjusted mean difference vs. controls); SE = standard error of β.

RMSSD = root mean square of successive differences, SDNN = standard deviation of NN intervals, pNN50 = percentage of successive intervals differing >50 ms; LF = low-frequency power; HF = high-frequency power; n.u. = normalized units; Δ = standing − supine

Spectral measures (LF, HF) log-transformed before regression.

Bold p-values indicate statistical significance (p < 0.05).

# Supplementary Table S2

| **ID** | **Group** | **Age** | **Sex** | **Supine SBP (mmHg)** | **Supine DBP (mmHg)** | **ΔSBP (mmHg)** | **ΔDBP (mmHg)** | **ΔHR (bpm)** | **SBP fall ≥20 mmHg** | **DBP fall ≥10 mmHg** | **ΔHR/ΔSBP ratio** | | **Treated AH** |
| --- | --- | --- | --- | --- | --- | --- | --- | --- | --- | --- | --- | --- | --- |
| Control_01 | Control | 56 | F | 121 | 100 | 4.2 | 14.4 | -7.8 | 0/4 | 4/4 | -1.82 | Yes | |
| Control_03 | Control | 22 | F | 110 | 95 | 2.0 | 14.2 | 14.5 | 0/4 | 3/4 | 7.25 | No | |
| Control_07 | Control | 54 | F | 124 | 74 | 33.0 | 4.0 | 27.2 | 4/4 | 0/4 | 0.83 | No | |
| Control_10 | Control | 62 | F | 161 | 84 | 31.0 | 4.2 | 10.5 | 4/4 | 0/4 | 0.34 | No | |
| Cohort_63 | CMT1A | 59 | F | 139 | 97 | 0.5 | 11.2 | 2.5 | 0/4 | 3/4 | 5.00 | Yes | |
| Control_36 | Control | 24 | M | 147 | 84 | 30.3 | 2.2 | -4.8 | 3/4 | 0/4 | -0.16 | No | |
| Control_44 | Control | 49 | F | 136 | 98 | 20.5 | -1.0 | -6.5 | 2/4 | 0/4 | -0.32 | Yes | |
| Control_47 | Control | 28 | M | 119 | 104 | -9.8 | 15.5 | 18.8 | 0/4 | 3/4 | -1.92 | No | |

All subjects meeting the 20/10 mmHg mean-based OH criterion (Freeman 2011).

ΔSBP / ΔDBP = supine − standing; positive = fall on standing. ΔHR = standing − supine; positive = rise on standing.

SBP fall ≥20 mmHg / DBP fall ≥10 mmHg: number of standing 1-min readings (out of 4) at which the threshold drop was observed.

ΔHR/ΔSBP ratio per Norcliffe-Kaufmann (2018); values <0.5 have been associated with neurogenic OH.

BP and HR were derived from the cuff-based 1-minute measurement protocol described in Methods and Materials.

AH = arterial hypertension

# Supplementary Table S3

| **Measure** | **Normal (n = 24)** | **Early CAN (n = 7)** | **Definite CAN (n = 4)** | **Spearman ρ (trend)** | **p (trend)** |
| --- | --- | --- | --- | --- | --- |
| Supine RMSSD (ms) | 30.6 | 22.9 | 10.3 | -0.39 | **0.022** |
| Supine SDNN (ms) | 28.9 | 21.6 | 11.1 | -0.41 | **0.015** |
| Supine pNN50 (%) | 6.6 | 2.5 | 0.0 | -0.39 | **0.021** |
| Supine HF power (ms²) | 281.0 | 106.7 | 65.4 | -0.39 | **0.021** |
| Supine LF power (ms²) | 328.6 | 236.2 | 69.3 | -0.38 | **0.026** |
| Supine HR (bpm) | 65.5 | 72.8 | 73.8 | 0.21 | 0.216 |

Medians shown for each Ewing CAN stage within the HRV-eligible CMT1A subgroup (n = 35).

Spearman ρ tests monotonic trend across the ordinal CAN stages (normal → early → definite).

Bold p-values indicate statistical significance (p < 0.05).

RMSSD = root mean square of successive differences, SDNN = standard deviation of NN intervals, pNN50 = percentage of successive intervals differing >50 ms; LF = low-frequency power; HF = high-frequency power; n.u. = normalized units; Δ = standing − supine

# Supplementary Table S4

| **Measure** | **CAN+ (n = 8)** | **CAN− (n = 16)** | **p** |
| --- | --- | --- | --- |
| **Ewing tests** | | | |
| E:I ratio | 1.1 (n=8) | 1.2 (n=16) | **0.002** |
| 30:15 ratio | 1.1 (n=8) | 1.4 (n=16) | **0.004** |
| Valsalva ratio | 1.9 (n=8) | 1.9 (n=16) | 0.830 |
| **HRV — supine** | | | |
| Supine RMSSD (ms) | 19.8 (n=8) | 33.0 (n=16) | **0.023** |
| Supine SDNN (ms) | 19.7 (n=8) | 37.6 (n=16) | **0.013** |
| Supine pNN50 (%) | 1.1 (n=8) | 13.7 (n=16) | **0.018** |
| Supine HF power (ms²) | 115.5 (n=8) | 460.7 (n=16) | **0.013** |
| Supine LF power (ms²) | 237.1 (n=8) | 672.3 (n=16) | 0.070 |
| Supine HR (bpm) | 63.0 (n=8) | 65.8 (n=16) | 0.150 |
| **HRV — standing** | | | |
| Standing RMSSD (ms) | 12.1 (n=8) | 23.4 (n=16) | **<0.001** |
| Standing SDNN (ms) | 16.0 (n=8) | 29.3 (n=16) | **<0.001** |
| **Other** | | | |
| CMTNSv2 | 9.0 (n=3) | 14.0 (n=10) | 0.236 |
| Age | 52.5 (n=8) | 40.5 (n=16) | 0.110 |

Median values per subgroup. p from Mann–Whitney U test (post-hoc subgroup comparison).

CAN+ defined as ≥1 abnormal Ewing test; CAN− = 0 abnormal tests.

Bold p-values indicate statistical significance (p < 0.05).

RMSSD = root mean square of successive differences, SDNN = standard deviation of NN intervals, pNN50 = percentage of successive intervals differing >50 ms; LF = low-frequency power; HF = high-frequency power; n.u. = normalized units; Δ = standing – supine; CMTNSv2 = CMT neuropathy scale version 2

# Supplementary Table S5

| **Patient ID** | **Variant (cDNA, NM_000166.6)** | **Codon** | **Cx32 domain** | **Sex** | **Age (yrs)** | **CMTNSv2** | **CAN stage** | **Abnormal cardiovascular tests** | **E:I ratio** | **30:15 ratio** | **Valsalva ratio** | **Supine RMSSD (ms)** | **Supine HF power (ms²)** | **Supine SBP/DBP (mmHg)** |
| --- | --- | --- | --- | --- | --- | --- | --- | --- | --- | --- | --- | --- | --- | --- |
| Cohort_42 | c.27G>T | 9 | N-terminus | F | 50 |  | Early CAN | E:I | 1.07 | 1.10 | 1.53 | 12.8 | 61 | 132/110 |
| Cohort_28 | c.88A>T | 30 | TM1 | M | 30 | 21 | Normal | — | 1.19 | 1.39 | 2.42 | 38.4 | 480 | 115/59 |
| Cohort_55 | c.184G>A | 62 | EC1 | F | 32 |  | Normal | — | 1.48 | 1.45 | 2.06 | 59.2 | 1842 | 118/64 |
| Cohort_43 | c.187G>T | 63 | EC1 | M | 40 | 26 | Normal | — | 1.26 | 1.21 | 2.27 | 21.8 | 248 | 161/109 |
| Cohort_11 | c.193T>C | 65 | EC1 | F | 59 |  | Early CAN | 30:15 | 1.16 | 1.06 | 1.56 | 28.5 | 230 | 102/76 |
| Cohort_68 | c.193T>C | 65 | EC1 | M | 32 |  | Normal | — | 1.34 | 1.13 | 1.52 | 17.7 | 153 | 140/94 |
| Cohort_53 | c.233C>T | 78 | TM2 | M | 48 | 13 | Normal | — | 1.30 | 1.36 | 1.40 | 57.2 | 1271 | 124/81 |
| Cohort_04 | c.287C>G | 96 | Cytoplasmic loop | F | 45 | 8 | Normal | — | 1.16 | 1.37 | 1.69 | 32.3 | 597 | 126/85 |
| Cohort_35 | c.287C>G | 96 | Cytoplasmic loop | F | 38 | 4 | Normal | — | 1.24 | 1.49 | 1.86 | 41.7 | 749 | 119/80 |
| Cohort_51 | c.473C>G | 158 | EC2 | F | 38 | 15 | Normal | — | 1.51 | 1.65 | 1.69 | 24.9 | 348 | 110/78 |
| Cohort_47 | c.491G>A | 164 | EC2 | M | 41 |  | Normal | — | 1.31 | 1.46 | 1.35 | 30.3 | 135 | 118/76 |
| Cohort_15 | c.541G>A | 181 | EC2 | M | 19 | 9 | Early CAN | 30:15 | 1.44 | 1.05 | 2.04 | 100.5 | 2755 | 103/68 |
| Cohort_16 | c.541G>A | 181 | EC2 | F | 45 | 7 | Early CAN | E:I | 1.11 | 1.10 | 1.87 | 16.3 | 73 | 107/82 |
| Cohort_18 | c.547C>T | 183 | EC2 | M | 33 | 12 | Normal | — | 1.24 | 1.64 | 2.68 | 55.1 | 688 | 106/77 |
| Cohort_19 | c.547C>T | 183 | EC2 | F | 59 | 11 | Early CAN | E:I | 1.11 | 1.32 | 2.76 | 28.2 | 155 | 123/80 |
| Cohort_26 | c.547C>T | 183 | EC2 | F | 57 |  | Early CAN | E:I | 1.07 | 1.16 | 1.99 | 22.0 | 99 | 116/96 |
| Cohort_36 | c.547C>T | 183 | EC2 | F | 58 | 7 | Normal | — | 1.21 | 1.27 | 2.29 | 30.2 | 258 | 128/86 |
| Cohort_10 | c.622G>A | 208 | TM4 | F | 55 |  | Early CAN | 30:15 | 1.14 | 1.06 | 1.96 | 15.0 | 76 | 144/91 |
| Cohort_37 | c.622G>A | 208 | TM4 | F | 45 | 16 | Normal | — | 1.21 | 1.35 | 1.91 | 33.7 | 300 | 102/82 |
| Cohort_41 | c.622G>A | 208 | TM4 | F | 52 |  | Normal | — | 1.19 | 1.34 | 1.79 | 32.3 | 441 | 114/80 |
| Cohort_65 | c.622G>A | 208 | TM4 | F | 32 |  | Normal | — | 1.59 | 1.24 | 1.90 | 52.9 | 1012 | 124/83 |
| Cohort_66 | c.622G>A | 208 | TM4 | M | 50 |  | Normal | — | 1.30 | 1.17 | 2.20 | 44.0 | 705 | 119/84 |
| Cohort_25 | c.643C>T | 215 | C-terminus | F | 47 | 17 | Normal | — | 1.23 | 1.09 | 1.40 | 18.4 | 99 | 132/85 |
| Cohort_32 | c.785_786del | 262 | C-terminus | F | 35 |  | Early CAN | E:I | 1.15 | 1.29 | 1.77 | 17.6 | 132 | 108/76 |

All variants heterozygous (females) or hemizygous (males) in GJB1 (NM_000166.6). Codon numbering refers to the encoded connexin 32 (Cx32) protein.

TM1 = transmembrane domain 1 ;EC1 = extracellular loop 1; EC2 = extracellular loop 2; TM4 = transmembrane domain 4, CMTNSv2 = CMT neuropathy scale version 2, RMSSD = root mean square of successive differences, HF = high-frequency power, SBP = systolic blood pressure, DBP = diastolic blood pressure

CAN staging: Normal = 0 abnormal cardiovascular autonomic reflex tests; Early CAN = 1 abnormal; Definite CAN = ≥2 abnormal; Severe CAN = Definite + OH (no CMTX1 patient met severe criteria).

Abnormal Ewing tests defined by age-stratified 2.3rd percentile cutoffs (Ziegler 1992); OH per Freeman 2011 (≥20/10 mmHg).
